# Use of a silver-coated plate to treat a postoperative infection after high tibial osteotomy – a case report

**DOI:** 10.5194/jbji-9-117-2024

**Published:** 2024-03-13

**Authors:** Rene Burchard, Jan A. Graw

**Affiliations:** 1 University of Marburg, Marburg 35037, Germany; 2 Department of Orthopaedics and Trauma Surgery, University Hospital of Giessen and Marburg, Marburg 35043, Germany; 3 Department of Orthopaedics and Trauma Surgery, Lahn-Dill-Kliniken, Dillenburg 35683, Germany; 4 Department of Anesthesiology and Intensive Care Medicine, Ulm University Hospital, Ulm 89081, Germany

## Abstract

Unilateral osteoarthritis of the knee can be treated by osteotomy. In case of postoperative infection after high tibial osteotomy, treatment can be challenging and often requires implant removal with the risk of loss of reduction.

In the presented case, a 47-year old patient suffered postoperative infection after high tibial osteotomy using an angular stable plate with the need for multiple revision surgeries and anti-infective therapy. Implant exchange to a silver-coated angular plate led to infection control with undisturbed wound healing and further bone consolidation. Full bone consolidation could be achieved radiographically 12 months after the last revision surgery. One-step implant exchange using silver-coated implants could be a promising approach to address postoperative infections after high tibial osteotomy.

## 1 Background

Unilateral osteoarthritis of the knee is a common pathology which can be treated either non-operatively with analgesics, intra-articular injections, or orthoses or by operative procedures including cartilage repair, osteotomies, or unicondylar endoprostheses (Burchard et al., 2018). In case of a deformity around the knee joint with pathological bony angles, osteotomies are the first choice of treatment. High tibial osteotomy (HTO) is an option for surgical therapy of a varus deformity. Since the evolution of internal plate fixators, open-wedge procedures with angular stable plate fixation have become a state-of-the-art fixation for HTO (Burchard et al., 2018).

With an incidence of up to 3 %–5 %, postoperative infections after HTO are rare but still a burden for patients and surgeons (Anagnostakos et al., 2013; Jia et al., 2023; Han et al., 2019). Several options exist to treat an implant-associated infection. Besides surgical debridement and implant retention with consecutive antibiotic drug application, implant exchange or even conservative treatment are possible alternatives (Anagnostakos et al., 2013).

Early postoperative infections at a stage when the osteotomy has not consolidated yet carry the risk of loss of reduction in the case of removal of the plate (Alt, 2017). Therefore, implant removal can result in a destabilized leg axis after open-wedge osteotomy and should be accompanied by additional fixation methods. In many cases, external fixation is applied. However, complete consolidation of an open-wedge HTO can need 12 to 18 months (Alt, 2017). Although a two-step procedure with a secondary to internal fixation after recovery from the infection is possible, there is still a risk of a recurring implant-associated infection (surgical site infection – SSI). For this reason, implants with antimicrobial activities would be useful. Silver-coated implants were developed to reduce the perioperative infection risk (Zajonz et al., 2017). So far, these implants have been used in periprosthetic joint infections and with an intramedullary nail for a fracture-related infection (Fiore et al., 2021; Alt, 2022). To the best of the authors' knowledge, no case of a silver-coated plate for the treatment of an implant-associated infection after a HTO has been reported.

## 2 Case report

**Figure 1:**
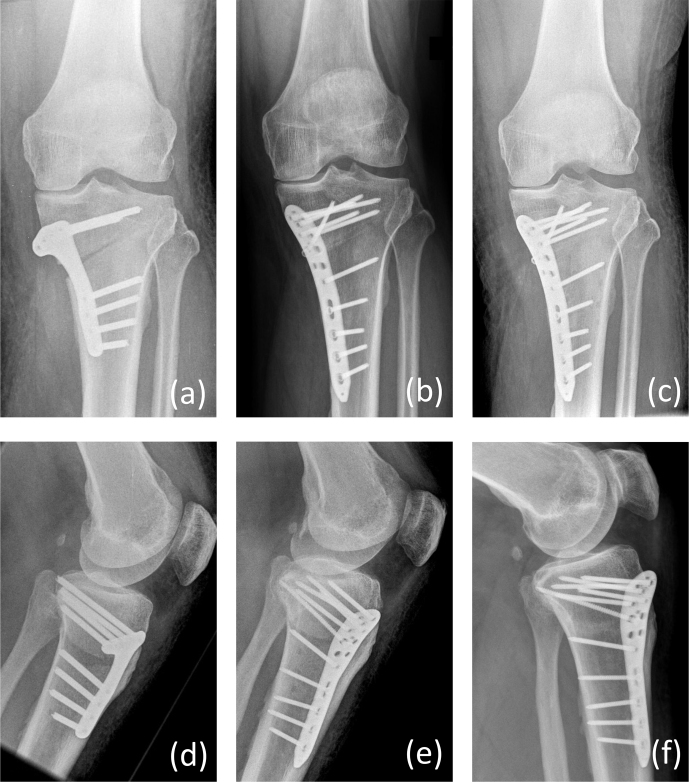
Anterior–posterior (a–p) radiograph on postoperative day 2 after primary osteotomy and osteosynthesis by a plate fixator **(a)**, radiograph on postoperative day 2 after implant exchange and osteosynthesis by a silver-coated plate fixator **(b)**, and radiograph 1 year after revision surgery with complete healing of the osteotomy **(c)**. Additionally, lateral views were shown in the same order as the (a–p) ones **(d–f)**.

A 47-year old male patient (no comorbidities, no former knee surgery, healthy vascular status) suffering from medial gonarthrosis combined with a pathological varus alignment and a corresponding medial tibial head angle of 84° was treated by HTO using an internal plate fixator (Activmotion S^®^, Size 2, Newclip GmbH, Augsburg, Germany). The procedure was performed by a board-certified senior surgeon under single-shot antibiotic protection by 1.5 g cefuroxime. Postoperative alignment met Fujisawa's point, and primary wound healing was given (Fig. 1a). The patient was instructed to maintain a 20 kg weight-bearing limit for 4 weeks. After a painless interval and irritation-free wound conditions, the patient presented with wound secretion and pain in the knee in postoperative week 7. C-reactive protein (CRP) was highly elevated with 19.5 mg dL^-1^ (reference < 0.5 mg dL^-1^). Due to the relatively short time of only 7 weeks after the initial procedure, a surgical revision procedure with implant retention and with antiseptic lavage (Serasept^®^, Serag-91, Wiessner GmbH, Naila, Germany) and for soft-tissue conditioning vacuum-assisted closure was performed. In addition, intravenous anti-infective therapy was started with ampicillin and sulbactame 3 g three times daily. Bacterial culture revealed the presence of multi-sensitive *Staphylococcus aureus*. After two surgical revision operations, wound closure was established and the patient left the hospital with oral antibiotic therapy with amoxicillin/clavulanic acid 875/125 mg and Rifampicin 450 mg, each two times daily for 6 weeks. Another 20 kg weight-bearing limit for 4 weeks was recommended. Two weeks later the infection recurred. Again, surgical revision with vacuum assistance was performed. Further options including external fixation or off-label use of a newly developed silver-coated plate (Loqtec^®^ antibacterial 3.5, aap Implantate AG, Berlin, Germany), which have not been officially Conformité Européenne (CE)-marked yet, were discussed. After informed consent, implantation of the silver-coated implant was chosen as a therapeutic approach, and the existing internal fixator was removed and exchanged by the silver-coated plate (Fig. 1b). After implant exchange the surgical wound healed uneventfully and the patient could be discharged to rehabilitation on postoperative day 6. Anti-infective therapy was again with amoxicillin/clavulanic acid and Rifampicin orally as used after the first revision surgery and for a period of 6 more weeks. A 20 kg weight-bearing limit was recommended for an additional 4 weeks. Laboratory infection parameters had returned to normal values. One year later, the osteotomy had healed completely, and the painless and satisfied patient returned to have HTO of his other leg (Fig. 1c).

## 3 Discussion

The presented case demonstrated a rare but severe complication after HTO. If implants are involved in infections, biofilms often develop after a few weeks (Donlan and Costerton, 2002). In these cases, one-step exchange of the implant entails the risk of an early recurrence of the biofilm (Pinto et al., 2020). Therefore, in the presented case, a silver-coated implant was chosen, and a favorable clinical result could be achieved.

Silver-coated implants were developed to prevent infection but not for treatment yet (Kuehl et al., 2016). Nevertheless, in this case, due to the recurrent SSI and the lack of suitable alternatives for the patient, it was decided to use the silver-coated plate systems, which are currently still under development, for off-label curative use. The off-label use was discussed in detail with the patient in advance, and the company was asked to provide the silver-coated implant.

However, there are several risks associated with the use of silver-coated implants, particularly the possible negative effect of silver on bone healing. The risk–benefit analysis was ultimately interpreted in favor of the implant use, also regarding experimental in vivo studies (Arens et al., 2020). However, in the case of implant-associated infection after HTO and a still unstable osteotomy after removal of the implant, this approach could be considered as an option.

Considering the positive findings in this singular case report, we eagerly await whether an ongoing observer-blinded randomized prospective study will demonstrate similarly beneficial results supporting general and broader use of this kind of silver-coated implant (Schoder et al., 2022).

## Data Availability

Data are available upon request to the corresponding author.
